# Analysis of IV Drugs in the Hospital Workflow by Raman Spectroscopy: The Case of Piperacillin and Tazobactam

**DOI:** 10.3390/molecules26195879

**Published:** 2021-09-28

**Authors:** Ioanna Chrisikou, Malvina Orkoula, Christos Kontoyannis

**Affiliations:** 1 Department of Pharmacy, University of Patras, University Campus, GR-26504 Rio Achaias, Greece; ioannach94@gmail.com (I.C.); malbie@upatras.gr (M.O.); 2 Institute of Chemical Engineering Sciences, Foundation of Research and Technology-Hellas (ICE-HT/FORTH), GR-26504 Platani Achaias, Greece

**Keywords:** medical errors, hospital workflow, patient safety, Raman spectroscopy, IV drugs, piperacillin, tazobactam, non-invasively

## Abstract

Medical errors associated with IV preparation and administration procedures in a hospital workflow can even cost human lives due to the direct effect they have on patients. A large number of such incidents, which have been reported in bibliography up to date, indicate the urgent need for their prevention. This study aims at proposing an analytical methodology for identifying and quantifying IV drugs before their administration, which has the potential to be fully harmonized with clinical practices. More specifically, it reports on the analysis of a piperacillin (PIP) and tazobactam (TAZ) IV formulation, using Raman spectroscopy. The simultaneous analysis of the two APIs in the same formulation was performed in three stages: before reconstitution in the form of powder without removing the substance out of the commercial glass bottle (non-invasively), directly after reconstitution in the same way, and just before administration, either the liquid drug is placed in the infusion set (on-line analysis) or a minimal amount of it is transferred from the IV bag to a Raman optic cell (at-line analysis). Except for the successful identification of the APIs in all cases, their quantification was also achieved through calibration curves with correlation coefficients ranging from 0.953 to 0.999 for PIP and from 0.965 to 0.997 for TAZ. In any case, the whole procedure does not need more than 10 min to be completed. The current methodology, based on Raman spectroscopy, outweighs other spectroscopic (UV/Vis, FT-IR/ATR) or chromatographic (HPLC, UHPLC) protocols, already applied, which are invasive, costly, time-consuming, not environmentally friendly, and require specialized staff and more complex sample preparation procedures, thus exposing the staff to hazardous materials, especially in cases of cytotoxic drugs. Such an approach has the potential to bridge the gap between experimental setup and clinical implementation through exploitation of already developed handheld devices, along with the presence of digital spectral libraries.

## 1. Introduction

In a hospital workflow routine, the intravenous administration of drugs to patients is a usual phenomenon. Some of the most important advantages of intravenous drug administration include the immediate and fast therapeutic result, which is of utmost importance in case of emergency and the treatment of patients who are unable to receive oral administration [1].

The errors that are associated with the preparation and administration of intravenous drugs constitute one of the most usual categories of medical errors, which can lead not only to morbidity but also to mortality due to the direct effect they have on patients. Up to date, a large number of studies have been conducted regarding the detection of intravenous medication errors. The majority of them have focused on the preparation and administration procedure and the frequency the errors occurred [2,3,4,5,6,7,8,9,10,11,12,13].

Two worth mentioning, typical examples of individual incidents of IV errors, include the unsuccessful identification of the drug administered and the administration of an incorrect drug dose. In the first case, epinephrine was administered instead of midazolam as anesthetic, due to the similarity in size and color of packaging between the two formulations, in a health unit in Egypt [14]. In the second case, an excessive paracetamol dose was administered to a three-month-old baby at a pediatric department of a university hospital in Turkey. The drug quantity finally administered was ten-fold larger than the desired, which caused transient hepatotoxicity to the infant [15].

Obviously, there is a need for measures to be taken in order for the above-mentioned medication errors, mainly those associated with the wrong drug or the wrong concentration–dose of it, to be prevented. The present study aims at proposing a suitable methodology, for identification and quantification of IV drugs. The proposed methodology should be non-invasive (without removal of the substance from the commercial glass bottle, the infusion bag, or the intravenous infusion set used in each stage of administration process), aseptic, non-destructive for the sample, and simple. Additionally, it should be fast and effective as it should be applied in a hospital workflow in cases of emergency, as well as of low cost so that it can be applied on a large scale. A possible candidate is Raman spectroscopy.

In the medical and biomedical field, Raman spectroscopy lists numerous applications, including oncologic (cancer diagnosis and monitoring), cardiovascular, and neurological (Alzheimer disease) developments [16,17,18,19]. In recent years, and regarding the hospital context, the potential of Raman spectroscopy to bridge the distance between bench and bedside has been emphasized. More specifically, Bourget and collaborators determined the concentration of three hospital antineoplastic drugs, 5-fluoruracil in elastomeric portable infusion pumps, so as to develop an analytical tool for geometrically complex therapeutic objects [20], and andriamycin and epirubicin in polyethylene syringes [21]. Le et al. used a handheld Raman device for the analysis of two isomeric anticancer drugs, doxorubicin and epirubicin, by direct measurement of the substances in containers [22], while Amin and his team validated a method for the determination of ganciclovir, a cytotoxic anticancer agent [23]. The analysis of the latter was performed directly, through sealed glass vials. In two other studies, five antineoplastic drugs (5-fluorouracil, gemcitabine, cyclophosphamide, ifosfamide, and doxorubicin), as well as three taxane drugs (cabazitaxel, docetaxel, and paclitaxel) were packaged in glass vials and then measured [24,25,26]. In the case of taxane drugs, a handheld spectrometer had been used. Furthermore, Makki et al. investigated the identification of four chemotherapeutic drugs (doxorubicin, daunorubicin, ifosfamide, and methotrexate) by means of Raman spectroscopy and the macroanalysis was performed from solutions placed in quartz cuvettes [27]. Lastly, Makki and his team analyzed solutions of three hardly distinguishable isomers, doxorubicin, daunorubicin, and epirubicin, which were placed in quartz cuvettes once more [28].

This paper reports on the employment of Raman spectroscopy for the development of a new methodology, aiming at the identification and quantification of a piperacillin (PIP) and tazobactam (TAZ), intravenously administered formulation, during the preparation process and before administration to the patient. The current methodology was developed with a view to being implemented in the clinical environment and applied to IV drugs, extensively, irrespective of the category to which they belong (antibiotic, chemotherapeutic, etc.). The drug used in the current study is an injectable formulation, in the form of powder for reconstitution, in which the mass ratio between the two APIs is equal to 8:1 (89% PIP/11% TAZ). Piperacillin is a broad-spectrum b-lactam antibiotic, which inhibits the synthesis of the bacterial cell wall, while tazobactam is a beta-lactamase inhibitor, which increases and expands the antimicrobial spectrum of piperacillin. In particular, it protects the antibiotic from degradation caused by beta-lactamase enzymes [29,30,31]. Up to date, in most cases, PIP and TAZ in formulations have been simultaneously analyzed through exploitation of time-consuming LC (liquid chromatography) protocols [32,33,34], a procedure that is required by pharmacopoeia for the identification of each API and its impurities [35]. Ultra-high-performance liquid chromatography tandem mass spectrometry (UHPLC–MS/MS) has also been applied for the simultaneous determination of the two APIs, not in the formulation but in different biological matrices (serum, urine, renal replacement therapy effluent) in regard to therapeutic drug monitoring (TDM) after administration [36,37]. Although this method requires microsample volumes and the run time does not exceed 5 min, the analytical procedure requires more expensive equipment and more specialized staff and cannot be applied directly to the as-received initial dry formulation before reconstitution. Furthermore, another vibrational technique, FT-IR/ATR spectroscopy, has been successfully applied for the identification and quantification of the two APIs in the commercial mixture. The methodology developed is more than promising for use in the clinical workflow; however, the whole procedure is partially invasive and acquires some more time to be completed [38]. UV/Vis spectroscopy has also been used, but its application proved to be complex since it requires selection of appropriate derivative order and smoothing factor [39,40]. One additional obstacle for the UV/Vis application in the current study is that extensive dilution of the drug solutions (far from the clinically relevant concentrations) is required, thus rendering such a process more prone to errors.

Taking all the above into consideration, the novelty of the current study lies in that there is no previous report on an analytical Raman methodology developed to be fully harmonized with the preparation and administration procedures of IV drugs in a clinical environment. Besides that, no known effort has been made for the simultaneous analysis of two APIs in the same formulation, which is in form of powder intended for reconstitution and not a solution, in both solid and liquid state. The results’ subsections following involve:

- The non-invasive analysis of the solid formulation (powder) in its glass vial before reconstitution,
- The non-invasive analysis of the reconstituted liquid formulation in its glass vial, and
- The analysis of the final (further diluted in the IV infusion bag in order to lie in the therapeutic concentration range) formulation just before administration; either it is placed in the drip chamber of the IV infusion set used (on line analysis) or in a Raman optic cell. The latter would be filled with 1–2 mL of the liquid drug, removed from the IV bag using an aseptic syringe (at-line analysis).

## 2. Results

### 2.1. Analysis of the Non-Reconstituted Solid Formulation through the Commercial Glass Bottle

#### 2.1.1. Identification of APIs

**Figure 1:**
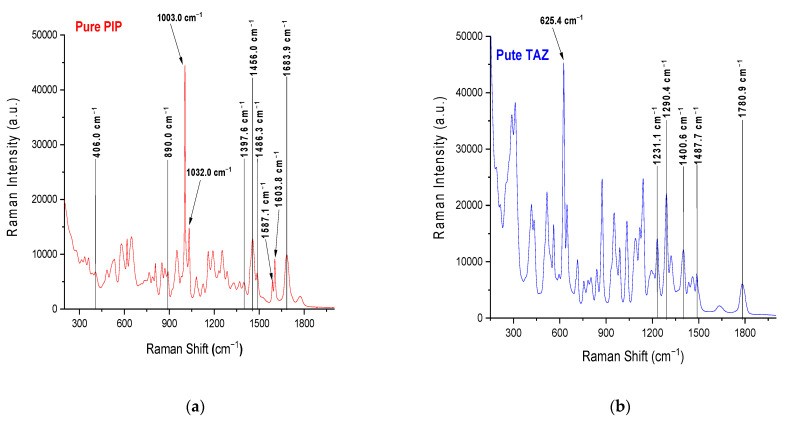
(**a**) Raman powder spectrum of pure piperacillin (PIP) and (**b**) Raman powder spectrum of pure tazobactam (TAZ). The peaks highlighted are the ones that are attributed to vibration modes in bibliography. The samples were placed on a g.slide.

**Figure 2:**
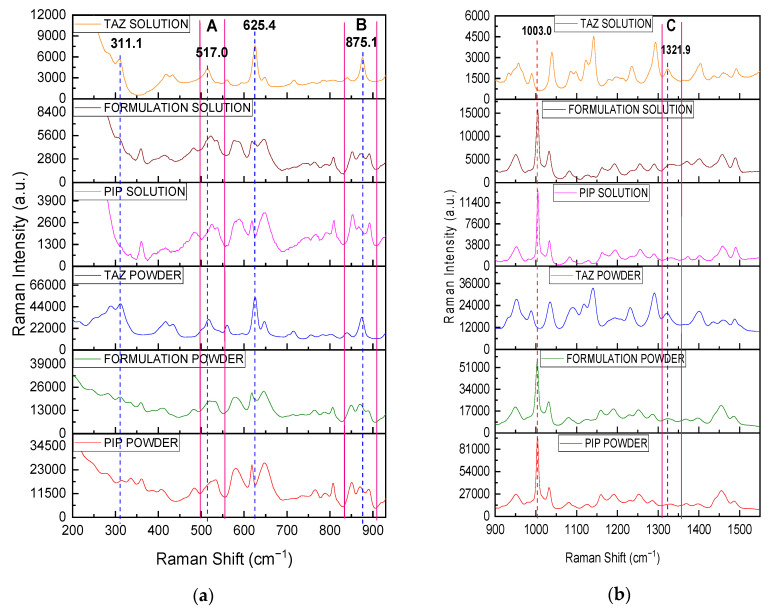
(**a**) Raman Spectra of PIP, TAZ, and the formulation, in both solid and liquid state: (**a**) from 200 to 930 cm^−1^ and (**b**) from 900 to 1550 cm^−1^. The samples were placed on a g.slide. Differences between PIP and formulation spectra due to TAZ presence in the commercial product are shown with blue dotted lines and in the spectral regions A (494–551 cm^−1^), B (826–906 cm^−1^), and C (1307–1355 cm^−1^). The characteristic peak of PIP at 1003 cm^−1^ is at the red dotted line.

Raman spectra of pure APIs and the formulation (mixture of PIP and TAZ), in both solid and liquid state, were obtained using a high reflectiveness gold-coated slide (g.slide) as a sampleholder (Figure 1 and Figure 2). Characteristic peaks of each API were detected and attributed to vibration modes of their molecules, according to the bibliography (Table 1) [41,42,43,44,45,46,47,48,49,50], and the formulation peaks were successfully attributed to the APIs without being shadowed by glass peaks.

A great similarity between the spectrum of the formulation and that of PIP is expected due to its high content in the mixture, while TAZ peaks are extensively overlapped by those of PIP, and its presence becomes mainly evident through small differences between formulation and PIP spectra, such as strengthening of PIP peaks at certain positions, broadening of certain peaks, and consequently, a slight shift of their center along the x axis or change in intensity ratio between neighboring PIP peaks. All these observations are summarized in Table 2.

**Figure 3:**
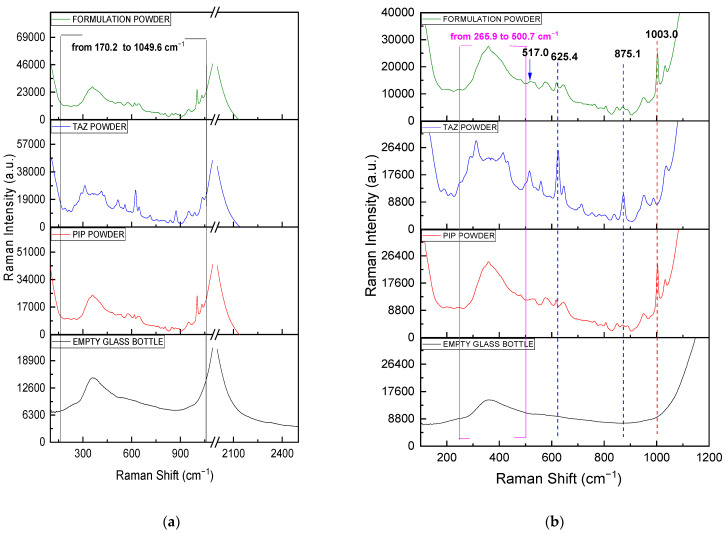
(**a**) Raman powder spectra of PIP, TAZ, and the formulation, obtained through the glass container of the formulation, as well as that of the empty glass bottle (blank sample): (**a**) from 100 to 2500 cm^−1^ and (**b**) from 100 to 1200 cm^−1^. The framed spectral region from 170 to 1050 cm^−1^ (black frame on the left) corresponds to the wavenumber range where the sample peaks appear. The framed spectral region from 266 to 500 cm^−1^ (pink frame on the right) corresponds to the wavenumber range where the sample peaks can hardly be detected, mainly in case of PIP and formulation powder, as they are shadowed by a broad glass peak. Differences between PIP and formulation spectra due to TAZ presence in the commercial mixture are shown with blue dotted lines and the blue arrow at 517 cm^−1^. The characteristic peak of PIP at 1003 cm^−1^ is at the red dotted line.

Subsequently, the Raman spectrum of the solid formulation was obtained without removing it from the glass bottle (non-invasively) and it is presented along with pure solid APIs’ spectra recorded through the glass container, as well as the spectrum of the empty bottle, which plays the role of the blank sample (Figure 3). All measurements were performed using the fiber optic probe of the Raman instrument, which was constantly in touch with the outer surface of the glass bottle, from six different marked regions of the latter. The commercial glass bottle had a diameter of 4.60 cm, while the mean thickness value coming from the six measuring positions was equal to 2.40 mm, with a standard deviation value of 0.19 mm.

Analytes’ peaks are detected in the spectral region ranging from 170 to 1050 cm^−1^ (Figure 3a, black framed region). The wavenumber range that has been cut out from the spectra is that between 1100 and 2000 cm^−1^, where a very broad and intense glass peak appears, masking any of the sample peaks. Once more, the great similarity between the spectrum of the formulation powder and that of PIP powder is clearly observed. Contrary to the observations made when the spectra were obtained with the g.slide, the small differences between PIP and formulation spectra due to TAZ presence are in this case limited to the bands at 517, 625, and 875 cm^−1^ (Figure 3b), while the difference at 311 cm^−1^ cannot be detected, as the sample peaks between 266 and 500 cm^−1^ are shadowed by a relatively broad glass peak (Figure 3b, pink framed region). The characteristic PIP peak at 1003 cm^−1^ is effectively detected once more.

#### 2.1.2. Quantification of APIs

Standard mixtures of the APIs, with a mass ratio ranging from 10% TAZ/90% PIP to 30% TAZ/70% PIP were prepared and measured after they had been placed in the glass bottle. The methodology included external usage of the fiber optic probe of the instrument, and six measurements were collected from different positions of the glass bottle. For the quantification of PIP, its characteristic peak at 1003 cm^−1^ was used. As for TAZ, no characteristic peak of it could be detected in the solid state. Nonetheless, after a variety of quantification efforts, the peak at 517 cm^−1^ proved to be the most appropriate for that purpose.

Worth mentioning is the fact that, as TAZ concentration in the standard solid mixtures increased, the intensity enhancement at 517 cm^−1^ became gradually more evident, and it ended up changing the morphology of the already existing PIP peak. The mean peak height ratio, *I*(517)/*I*(1003) and relative standard deviation (RSD) values [51] resulting from the six replicate measurements were calculated for each standard mixture (Table 3).

- Linearity

A minimum of five concentrations is required for the establishment of linearity, according to ICH guidelines for validation of analytical methods [52]. In this case, the linearity of the method was confirmed by a five point concentration calibration curve, achieved by preparing a series of separate weighings of synthetic mixtures of the drug product components.

**Figure 4:**
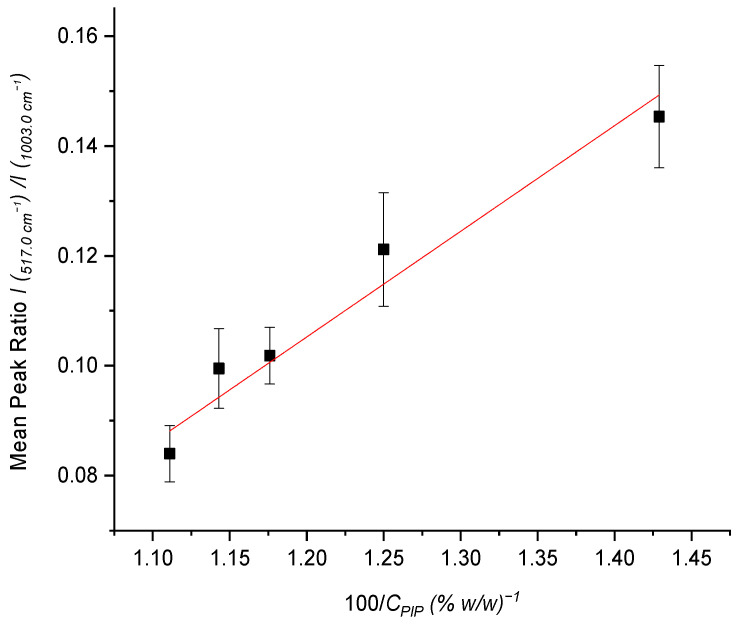
Calibration line for both PIP and TAZ for standard solid mixtures of them, with APIs’ mass ratio ranging from 10% TAZ/90% PIP to 30% TAZ/70% PIP.

The calibration graph plotted is reproduced in Figure 4 and was constructed through least square regression method, using the ratio $\frac{I\left(517\right)}{I\left(1003\right)}$ (y axis) against ratio $\frac{100}{C_{PIP}}$ (x axis) (see Appendix A).

The equation describing the calibration line was:

(1) $$\frac{I\;\left(517\;{\mathrm{cm}}^{-1}\right)}{I\;\left(1003\;{\mathrm{cm}}^{-1}\right)}=(0.192\;\pm \;0.025)\times \left(\frac{100}{C_{PIP}}\right)+(-0.125\;\pm \;0.029),\;(R^{2}=0.953)$$

- Intra- and Inter-day Precision

The intra-day (repeatability) and inter-day precision of the developed method were assessed using 6 determinations for each, at 100% of the test concentration, i.e., 11% TAZ/89% PIP (commercial drug product), characterized by a × (100/*C_PIP_*) value of 1.124. The repeatability study results gave a 100/ *C_PIP_* value of (1.108 ± 0.041) (% *w*/*w*)^−1^, with a RSD value equal to 3.70%. Adiditionally, treating the precision test sample as unknown, PIP and TAZ concentration values, along with their total error [53], were found to be (90.17 ± 1.95) % *w*/*w* and (9.83 ± 1.95) % *w*/*w*, respectively. The relative error values (E_r_) of the concentrations determined above were equal to 1.31% and 10.64%, respectively [54].

Accordingly, the inter-day precision study, tested over a period of 6 days, gave a x value of (1.110 ± 0.042) (% *w*/*w*)^−1^*,* with a RSD value equal to 3.76%.

- Accuracy

The accuracy of the method was assessed using nine determinations over three concentrations (three determinations for each concentration), covering the specified range [52]. The results gave:

1. For 30% TAZ/70% PIP, x = (1.410 ± 0.070) (% *w*/*w*)^−1^, RSD = 4.68%, and E_r_ = 1.33%.
2. For 15% TAZ/85% PIP, x = (1.189 ± 0.039) (% *w*/*w*)^−1^, RSD = 3.32%, and E_r_= 1.07%
3. For 10% TAZ/90% PIP, x = (1.080 ± 0.026) (% *w*/*w*)^−1^, RSD = 2.38%, and E_r_= 2.80%.

- LoD and LoQ

Since a minimum amount of tazobactam corresponds to a maximum amount of piperacillin, the limit of detection (LοD) of tazobactam was calculated after many blank (PIP powder) measurements [55]. Using Equation (1), LοD was found to be 3.01% *w*/*w* and LοQ (limit of quantification) equal to 9.03% *w*/*w*. The LοD value was confirmed by visual evaluation method [52].

- Calibration Range

The concentration range used for the construction of the calibration line corresponds to values from 10% TAZ/90% PIP to 30% TAZ/70% PIP, within which analytical procedure provides an acceptable degree of linearity, precision, and accuracy. The lower limit of the calibration range could not be less than 10% TAZ/90% PIP, as it is conformed to the LoQ value of TAZ API.

### 2.2. Analysis of the Reconstituted Formulation through the Commercial Glass Bottle

#### 2.2.1. Identification of APIs

**Figure 5:**
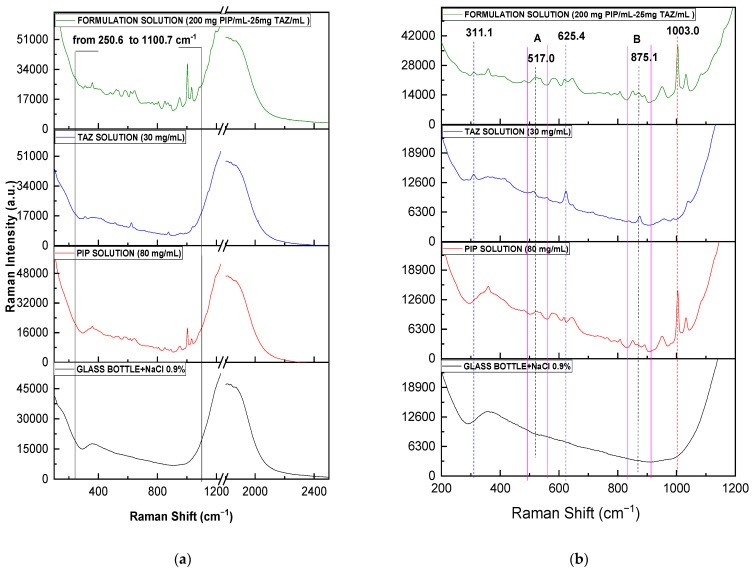
(**a**) Raman solution spectra of PIP (80 mg/mL), TAZ (30 mg/mL), and the formulation (200.00 mg PIP/mL and 25.00 mg TAZ/mL), obtained through the glass container of the formulation, as well as that of the glass bottle filled with NaCl 0.9% (blank sample): (**a**) from 100 to 2500 cm^−1^ and (**b**) from 200 to 1200 cm^−1^. The framed spectral region from 250 to 1100 cm^−1^ (black frame on the left) corresponds to the wavenumber range where the sample peaks appear. Differences between PIP and formulation spectra due to TAZ presence are with blue dotted lines and in the spectral regions A (493–562 cm^−1^) and B (834–913 cm^−1^). The characteristic peak of PIP at 1003 cm^−1^ is with the red dotted line.

Adhering to the instructions of the usage leaflet of the product, 20 mL of NaCl 0.9% were used for the complete reconstitution of the solid formulation in the commercial glass bottle. According to calculations, the reconstituted sample had a nominal concentration of 200.00 mg PIP/mL and 25.00 mg TAZ/mL. Subsequently, its Raman spectrum was obtained without removing it out of the glass bottle (non-invasively) and was compared to spectra from pure APIs’ solutions (80 mg PIP/mL and 30 mg TAZ/mL) that had been measured in the same way, i.e., using the fiber optic probe of the Raman instrument (Figure 5).

As it was expected, a great similarity is noticed between PIP and formulation spectrum, while above 1100 cm^−1^, sample peaks are shadowed by the broad glass peak appearing. The characteristic PIP peak at 1003 cm^−1^ (vertical dashed red line) is highly detectable, while TAZ presence in the liquid formulation is confirmed through four different spectral positions (vertical dashed blue lines), at which the formulation spectrum is differentiated from that of pure PIP. Particularly, three of the four above-mentioned positions (517 (area A), 625, and 875 (area B) cm^−1^) had been highlighted in Figure 3, which includes the powder spectra of the respective samples placed in the glass bottle. Furthermore, another peak at 311 cm^−1^ is clearly noticed, which, in this case, seems to be characteristic of TAZ API (see Figure 2 and Table 2).

#### 2.2.2. Quantification of APIs

The reconstitution of the solid formulation in the commercial glass bottle was performed twice, once for preparing the concentrated, first standard solution of PIP with a nominal concentration of 266.67 mg/mL and a second time for preparing the respective solution for TAZ, with a nominal concentration of 60.00 mg/mL. Their Raman spectra were then obtained, in the same way as has already been described. By the method of successive dilutions for each of the concentrated solutions, another 10 standards were prepared, and their nominal concentration values ranged from 200.00 to 5.00 mg PIP/mL, as well as nine more standards with nominal concentration values ranging from 50.00 to 7.00 mg TAZ/mL. Each one of them was measured before it was further diluted.

Each API was quantified separately, using the peak at 1003 cm^−1^ for PIP and that at 311 cm^−1^ for TAZ. For every standard solution, the height/intensity value for both selected peaks was calculated for all of the six marked areas of the glass bottle (baseline correction method), after subtraction of the glass contribution to the final result for each area separately. The mean peak height values (*I1003(sample)* − *I1003(blank)*, *I311(sample)* − *I311(blank)*) along with the relative standard deviation values (RSD) were then calculated from the six replicate measurements performed (Table 4).

**Figure 6:**
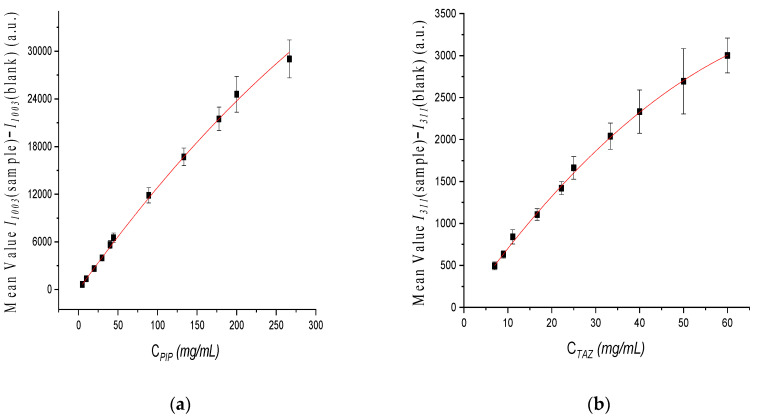
Calibration curve of: (**a**) PIP and (**b**) TAZ, after polynomial fitting of the points for the whole concentration range.

Calibration curves, one for each API, were constructed using the intensity values (y axis) against concentration values (x axis). Particularly, for PIP quantification, the value *I1003(sample)* − *I1003(blank)* was used against *C_PIP_* and for TAZ quantification *I311(sample)* − *I1311(blank)* against *C_TAZ_*, respectively. The linear fitting of the standard points for the whole concentration range tested was not effective, proving that there is not a single straight line that can appropriately express the relationship between API concentration and Raman intensity. This was expected, taking into consideration the solubility values of PIP and TAZ, which are equal to 50 mg/mL for both APIs [56,57], i.e., part of each API was not dissolved using the reconstitution instructions of the manufacturer (see Section 2.2.2). Polynomial fitting of the standard points gave much better results (Figure 6).

The respective equations describing the calibration curves resulting after polynomial fitting were:

*I1003(sample)* − *I1003(blank)* = (−0.10 ± 0.02) × (*C_PIP_)* ^2^+ (138.94 ± 2.88) × *C_PIP_* + (−40.82 ± 31.46), (R^2^ = 0.999) (2)

and

*I311(sample)* − *I311(blank)* = (−0.40 ± 0.06) × (*C_TAZ_*)^2^+ (73.99 ± 3.07) × *C_TAZ_* + (1.35 ± 28.86), (R^2^ = 0.997) (3)

- Intra- and Inter-day Precision

The intra-day (repeatability) and inter-day precision, tested over a period of 6 days, of the developed method were assessed using six determinations for each at 100% of the test concentration directly after the reconstitution process, i.e., 200.00 mg PIP/mL and 25.00 mg TAZ/mL.

For PIP API, Equation (2) was used and the repeatability study results gave: C = (217.38 ± 17.92) mg/mL and RSD = 8.24%. The inter-day precision gave: C = (218.07 ± 18.02) mg/mL and RSD = 8.26%.

For TAZ API Equation (3) was used and the repeatability study results gave: C = (26.18 ± 2.52) mg/mL and RSD = 9.62%. The inter-day precision study resulted in: C = (27.23 ± 2.48) mg/mL and RSD = 9.12%.

- Accuracy

The accuracy of the method was assessed using 9 determinations over 3 concentration (3 determinations for each concentration), covering the specified range for each API. The accuracy test for PIP gave:

1. For 266.67 mg/mL, C = (264.12 ± 25.19) mg/mL, RSD = 9.54% and E_r_ = 0.96%.
2. For 44.44 mg/mL, C = (45.02 ± 1.23) mg/mL, RSD = 2.74% and E_r_ = 1.31% and
3. For 5.00 mg/mL, C = (5.02 ± 0.33) mg/mL, RSD = 6.66% and E_r_ = 0.40%.

For TAZ API, was respectively:

1. For 60.00 mg/mL, C = (63.70 ± 6.22) mg/mL, RSD = 9.76% and E_r_ = 6.17%.
2. For 33.33 mg/mL, C = (35.55 ± 3.02) mg/mL, RSD = 8.49% and E_r_ = 6.66% and
3. For 7.00 mg/mL, C = (7.31 ± 0.83) mg/mL, RSD = 11.37% and E_r_ = 4.43%.

- LoD and LoQ

Using Equation (2), and after many blank samples had been measured, PIP LoD was found to be 0.60 mg/mL, while LoQ was equal to 1.80 mg/mL. Respectively, using Equation (3), TAZ LoD was found to be 1.85 mg/mL, while LoQ was equal to 5.55 mg/mL. LoD values for both APIs were confirmed by visual evaluation method.

- Calibration Range

The concentration range used for the construction of the calibration curves corresponds to values from 5.00 to 266.67 mg/mL for PIP and from 7.00 to 60.00 mg/mL for TAZ, within which the analytical procedure provides an acceptable degree of precision and accuracy.

In order to test the performance of the polynomial calibration curves for each API once more, two different formulation solutions were prepared to be treated as unknown samples, and they were measured in the same way:

One solution containing 152.00 mg PIP/mL and 19.00 mg TAZ/mL. Using Equation (2), the PIP concentration was found to be (143.52 ± 10.52) mg/ mL with a relative error of 5.58%. Using Equation (3), the TAZ concentration was found to be (20.42 ± 1.42) mg/ mL with a relative error of 7.47%.

One solution containing 28.80 mg PIP/mL and 3.60 mg TAZ/mL. Using Equation (2), the PIP concentration was found to be (27.05 ± 2.02) mg/ mL with a relative error of 6.08%. Using Equation (3), the TAZ concentration was found to be (3.30 ± 0.75) mg/ mL with a relative error of 8.32%. Due to the fact that the TAZ concentration of the second unknown sample, i.e., 3.60 mg/mL, is below the LoQ value, and its quantification is more than satisfying, the robustness of the current methodology is confirmed, i.e., the validity of the analytical procedure is maintained regardless of small variations in the concentration range used.

Given the RSD values included in Table 3 and Table 4 and the glass-induced thickness variability of the commercial bottle, six measurements for each sample would be needed in case of non-invasive analysis of either the solid drug before reconstitution or the liquid drug after reconstitution. Thus, the measurement process would take approximately 10 min.

#### 2.2.3. Solubility of APIs after Reconstitution

In order to experimentally verify the incomplete dissolution of the solid formulation after reconstitution and quantify the percentage of drug that could not be diluted after reconstitution according to instructions, the solution being prepared was filtered. The filtrate was diluted five times in order for its concentration to be in the range applicable for Equations (2) and (3), while the filtering membrane was finally kept in a glass container with dehydrating material in order to remove the remaining moisture.

Subsequently, Raman spectra were obtained from ten different points/crystals of the remaining powder on the filter membrane, and the spectra found to be identical and compatible with the initial formulation powder, i.e., both APIs were identified in the spectra obtained from the undissolved material.

The filtrate that was diluted was then treated as an unknown sample and was measured in the same way as the standard solutions. Using Equations (2) and (3), it was found that the filtrate solution contained (33.79 ± 2.36) mg PIP/mL and (4.02 ± 0.66) mg TAZ/mL, respectively, i.e., approximately 15% of the initial solid formulation remained undissolved after the reconstitution process.

### 2.3. Analysis of the Liquid Formulation in the IV Bag

According to the instructions of use, 20 mL of the reconstituted formulation, including the undissolved APIs, is inserted aseptically in the IV infusion bag so as to be further diluted to a final volume of 100–150 mL. At that volume range, PIP concentration in the final solution will be between 26.67 and 40.00 mg/mL and that of TAZ between 3.33 and 5.00 mg/mL, i.e., both APIs will be completely dissolved. After the final preparation of the IV solution, the infusion bag content can be analyzed, either at-line or on-line. The at-line analysis requires the removal with an aseptic syringe of 1–2 mL of the solution and usage of an external optic Raman cell. The on-line analysis instead, which is preferable, requires the non-invasive analysis of the liquid in the IV drip chamber, a device that is used to allow air to rise out from a fluid so that it is not passed downstream, using Raman fiber optic.

#### 2.3.1. Quantification of APIs through the Raman Optic Cell (At-line Analysis)

**Figure 7:**
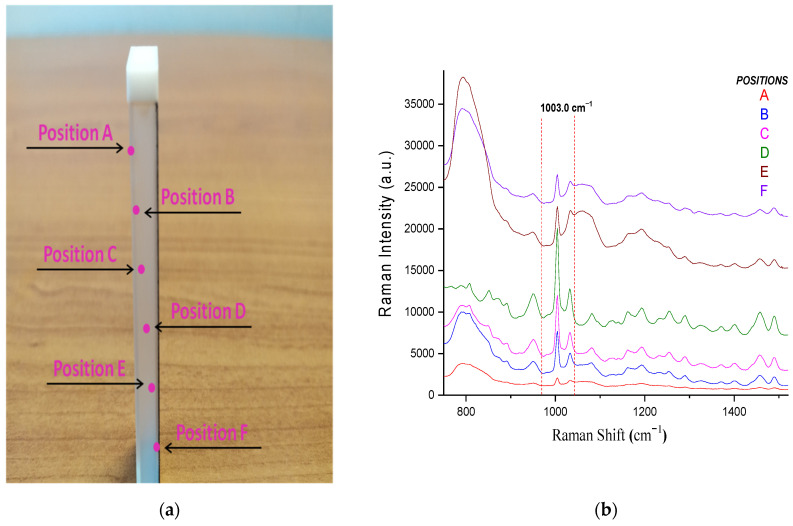
(**a**) Schematic illustration of the cross-section of the Raman cell depicting the depth of the six different focus positions of the laser beam, starting from the external quartz surface to the mirror surface for the spectra acquisition (for better clarity, each position of the focused beam is depicted along the diagonal of the cell), (**b**) Raman spectra of the liquid formulation (40.00 mg PIP/mL and 5.00 mg TAZ/mL) placed in the cell, each one corresponding to the radiation focus positions illustrated in part (**a**) of the figure.

The Raman optic cell constitutes a special quartz cuvette with a mirror placed on the back part of it. The radiation path in the cell corresponds to a distance of 5 mm, while either the front quartz or the back mirror wall has a thickness of 1mm. In order to establish the ideal focus distance of the fiber optic probe on the Raman cell, a variety of focus positions of the incident laser beam were tested (Figure 7a). The cell was filled with a formulation solution of 40.00 mg PIP/mL and 5.00 mg TAZ/mL. The determining factor for the selection of the most appropriate focus position of the radiation was the intensity of the predominant, characteristic PIP peak at 1003 cm^−1^ (Figure 7b).

In Table 5, comments can be found on the focus positions tested for the Raman optic cell, as well as on the quality of the respective spectrum received each time, with the latter being the determining factor for choosing the most appropriate position.

Based on Figure 7b, the laser beam should be focused on the D position, i.e., close to the mirror surface, which provides the strongest Raman signal, and thus all the following Raman spectra reported in the Raman cell section were obtained from the optimum position.

**Figure 8:**
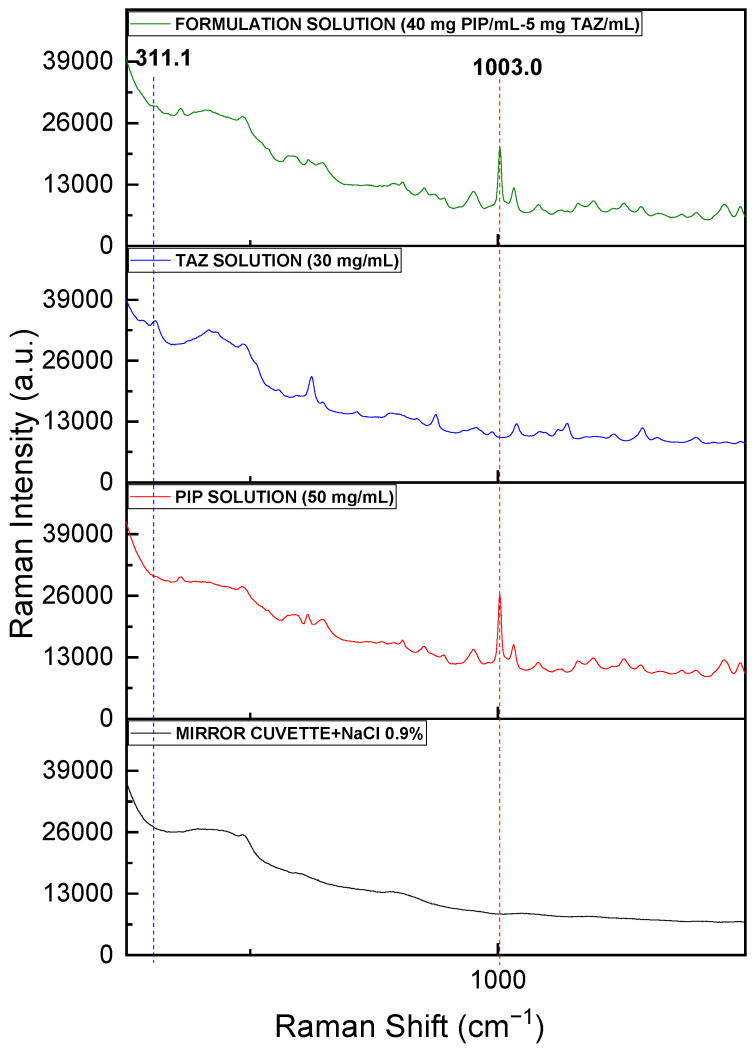
Raman spectra for solutions of pure PIP (50 mg/mL), pure TAZ (30 mg/mL), and the formulation (40.00 mg PIP/mL and 5.00 mg TAZ/mL), after the samples had been placed in the optic cell, as well as the blank sample spectrum (cell + NaCl 0.9%). PIP peak at 1003 cm^−1^ is highlighted by the red dashed line, whereas TAZ peak at 311 cm^−1^ is at the blue one.

The spectrum of the liquid formulation (40.00 mg PIP/mL and 5.00 mg TAZ/mL), acquired through the Raman optic cell using the fiber optic probe, was compared to the spectra of pure APIs’ solutions (50.00 mg PIP/mL and 30.00 mg TAZ/mL respectively) and that of the blank sample, which, in this case, is the optic cell filled with saline solution (NaCl 0.9%). Characteristic peaks of each API, at 1003 cm^−1^ for PIP and at 311 cm^−1^ for TAZ, were detected, as shown in Figure 8.

It is apparent that the quartz material of which the cell is made, the thinner glass, and the vertical surfaces of the cell allow the detection of sample peaks in a wider spectral window compared to the common glass material of the commercial formulation bottle, which causes the extensive masking of them.

Standard solutions were prepared by the method of successive dilutions, and 1.7 mL of each one was transferred in the cell to be measured. The measurement was repeated three times for three different portions of the sample under test. The standards had concentration values that ranged from 44.44 to 5.00 mg/mL for PIP and 5.56 to 3.75 mg/mL for TAZ (therapeutic concentration range included).

Aiming at quantifying the APIs in the IV infusion bag used, the height/intensity values for both selected peaks (311 cm^−1^ for TAZ and 1003 cm^−1^ for PIP) were calculated for each standard solution, after baseline correction and subtraction of the quartz material contribution (resulting from a variety of blank measurements). The mean peak height values (*I1003(sample)* − *I1003(blank), I311(sample)* − *I311(blank)*) along with the relative standard deviation values (RSD) were then calculated from the three replicate measurements performed (Table 6).

- Linearity

The linearity of the method was confirmed by a six point concentration calibration curve for PIP and a five point concentration calibration curve for TAZ, achieved by successive dilutions of an initial drug solution.

**Figure 9:**
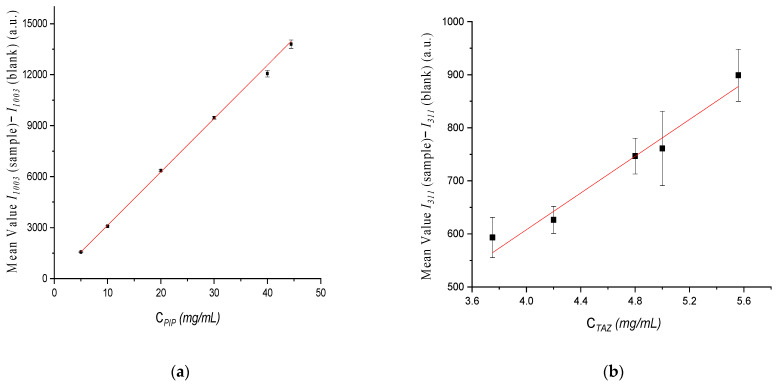
Calibration curve of: (**a**) PIP and (**b**) TAZ, for standard solutions of liquid formulation placed in the Raman optic cell before administration, with concentration values ranging from 44.44 to 5.00 mg/mL for PIP and from 5.56 to 3.75 mg/mL for TAZ respectively.

Calibration curves were constructed through least square regression method, using the intensity values (y axis) against concentration values (x axis) (Figure 9). Particularly, for PIP quantification the value *I1003(sample)* − *I1003(blank)* was used against *C_PIP_* and for TAZ quantification *I311(sample)* − *I1311(blank)* against *C_TAZ_* respectively.

The respective equations describing the calibration lines were:

*I1003(sample)* − *I1003(blank)* = (314.30 ± 3.81) × *C_PIP_* + (−8.38 ± 52.20), (R^2^ = 0.999) (4)

and

*I311(sample)* − *I311(blank)* = (173.05 ± 19.13) × *C_TAZ_* + (−84.21 ± 85.82), (R^2^ = 0.965) (5)

- Intra- and Inter-day Precision

The intra-day (repeatability) and inter-day precision, tested over a period of 6 days, of the developed method were assessed using six determinations for each at 100% of the test concentration, i.e., 40.00 mg PIP/mL and 5.00 mg TAZ/mL. 

For PIP API, Equation (4) was used, and the repeatability study results gave: C = (40.34 ± 1.79) mg/mL and RSD = 4.44%. The inter-day precision gave: C = (39.34 ± 1.28) mg/mL and RSD = 3.27%.

For TAZ API, Equation (5) was used, and the repeatability study results gave: C = (5.29 ± 0.44) mg/mL and RSD = 8.26%. The inter-day precision gave: C = (4.79 ± 0.49) mg/mL and RSD = 10.33%.

- Accuracy

The accuracy of the method was assessed using nine determinations over three concentrations (three determinations for each concentration), covering the specified range for each API. The accuracy test for PIP gave:

1. For 44.44 mg/mL, C = (43.93 ± 0.78) mg/mL, RSD = 1.77% and E_r_ = 1.15%.
2. For 20.00 mg/mL, C = (20.24 ± 0.15) mg/mL, RSD = 0.75% and E_r_ = 1.20% and
3. For 5.00 mg/mL, C = (5.00 ± 0.08) mg/mL, RSD = 1.65% and E_r_ = 0.00%.

For TAZ API, was respectively:

1. For 5.56 mg/mL, C = (5.68 ± 0.28) mg/mL, RSD = 5.00% and E_r_ = 2.16%.
2. For 4.80 mg/mL, C = (4.80 ± 0.20) mg/mL, RSD = 4.06% and E_r_ = 0.00%.
3. For 3.75 mg/mL, C = (3.91 ± 0.22) mg/mL, RSD = 5.61% and E_r_ = 4.27%.

- LoD and LoQ

Using Equation (4), and after many blank samples had been measured, PIP LoD was found to be 0.24 mg/mL, while LoQ was equal to 0.72 mg/mL. Respectively, using Equation (5) TAZ LoD was found to be 0.94 mg/mL, while LoQ was equal to 2.81 mg/mL. LoD values for both APIs were confirmed by visual evaluation method.

- Calibration Range

The concentration range used for the construction of the calibration lines corresponds to values from 5.00 to 44.44 mg/mL for PIP and from 3.75 to 5.56 mg/mL for TAZ, within which analytical procedure provides an acceptable degree of linearity, precision, and accuracy. Additionally, this range includes the therapeutic concentration window and ensures the total dissolution of the APIs present in the formulation intended for administration.

In order to test the performance of the calibration lines for each API once more, a formulation solution of 23.00 mg PIP/mL and 2.87 mg TAZ/mL was prepared in order to be treated as unknown sample and it was measured in the same way. Using Equation (4), PIP concentration was found to be (22.46 ± 0.60) mg/ mL with a relative error of 2.45%. Using Equation (5), TAZ concentration was found to be (3.05 ± 0.11) mg/ mL with a relative error of 6.27%. Given the low RSD values included in Table 6 and the advantages the Raman cell offers, regarding its construction, it would be satisfying one measurement to be performed, thus the whole procedure (withdrawal of the substance and measurement) would not take more than 5 min.

#### 2.3.2. Quantification of APIs through IV Drip Chamber (On-Line Analysis)

**Figure 10:**
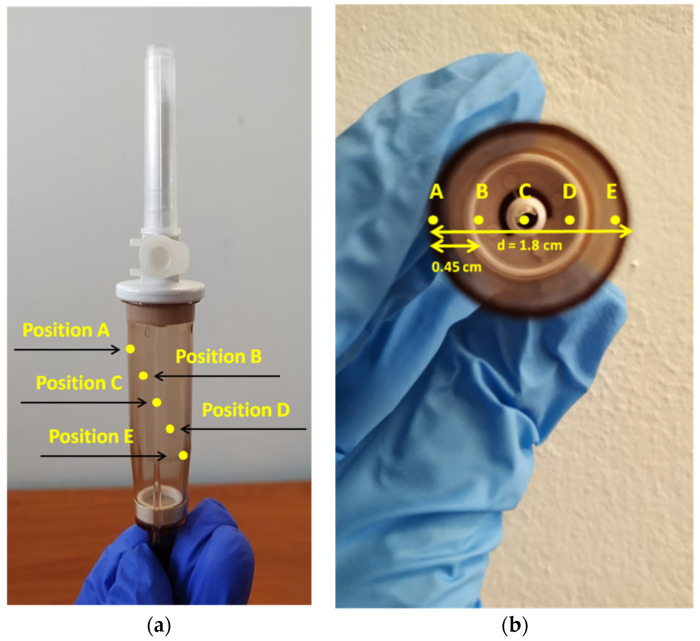
(**a**) Cross section schematic illustration and (**b**) top view of the drip chamber, along with the five different focus positions of the laser beam for the spectra acquisition. The diameter of the cylindrical drip chamber is equal to 1.8 cm, while the focus positions are equidistant, and the distance between them is 0.45 cm.

**Figure 11:**
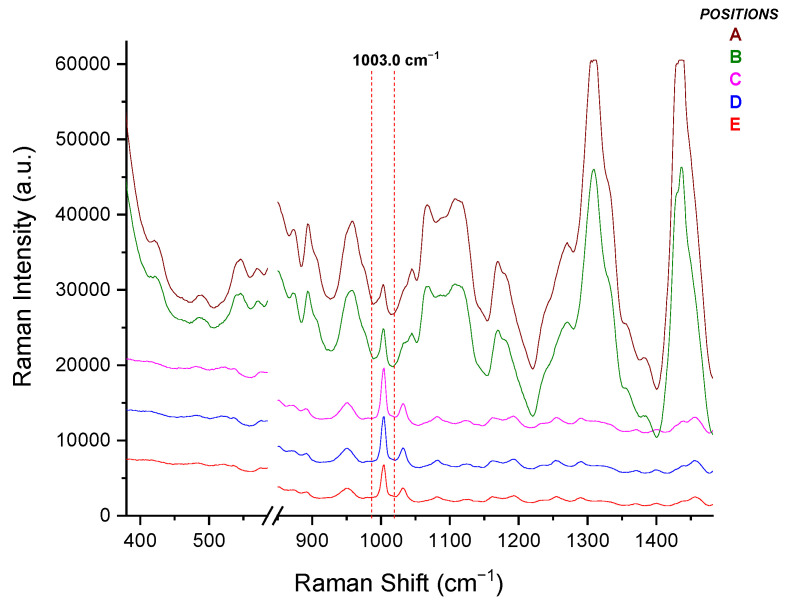
Raman spectra of the liquid formulation (40.00 mg PIP/mL and 5.00 mg TAZ/mL) placed in the drip chamber, each one corresponding to the radiation focus positions illustrated in Figure 10.

As in the case of the Raman optic cell, a variety of focus positions of the incident laser beam were tested (Figure 10) in order to establish the ideal focus position of the laser beam. The chamber was filled with a formulation solution of 40.00 mg PIP/mL and 5.00 mg TAZ/mL. The determining factor for the selection of the most appropriate focus position of the radiation was the intensity of the predominant, characteristic PIP peak at 1003 cm^−1^ (Figure 11).

In Table 7, comments can be found on the focus positions tested for the IV drip chamber, as well as on the quality of the respective spectrum received each time, with the latter being the determining factor for choosing the most appropriate position.

Based on Figure 11, the laser beam should be focused on the C position, which has yielded the most intense Raman signal, and thus all the following Raman spectra reported in the drip chamber section were obtained from the optimum position.

**Figure 12:**
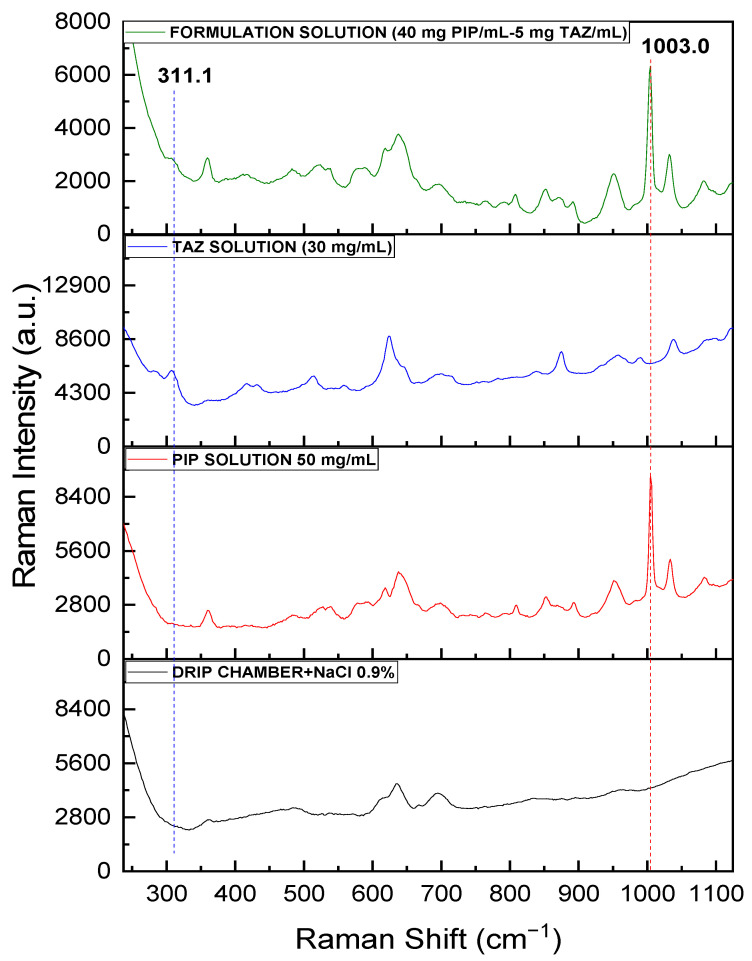
Raman spectra for solutions of pure PIP (50 mg/mL), pure TAZ (30 mg/mL), and the formulation (40.00 mg PIP/mL and 5.00 mg TAZ/mL), after the samples had been placed in the drip chamber, as well as the blank sample spectrum (drip chamber + NaCl 0.9%). PIP peak at 1003 cm^−1^ is highlighted with the red dashed line, whereas TAZ peak at 311 cm^−1^ with the blue one.

The spectrum of the liquid formulation (40.00 mg PIP/mL and 5.00 mg TAZ/mL), acquired through the drip chamber using the Raman fiber optic probe, was compared to the spectra of pure APIs’ solutions (50.00 mg PIP/mL and 30.00 mg TAZ/mL, respectively) and that of the blank sample, which, in this case, is the drip chamber filled with saline solution (NaCl 0.9%). Characteristic peaks of each API, at 1003 cm^−1^ for PIP and at 311 cm^−1^ for TAZ, were detected, as shown in Figure 12.

Although the plastic material, of which the drip chamber is made, contributes significantly to the obtained spectrum, the peaks attributed to the drip chamber are not overlapping significantly with API peaks.

Standard solutions were prepared by the method of successive dilutions and transferred in the drip chamber to be measured. The measurement was repeated three times for three different portions of the sample under test. The standards had concentration values that ranged from 44.44 to 5.00 mg/mL for PIP and 5.56 to 0.62 mg /mL for TAZ (therapeutic concentration range included).

Aiming at quantifying the APIs in liquid formulation using the current methodology, the height/intensity values for both selected peaks (311 cm^−1^ for TAZ and 1003 cm^−1^ for PIP) were calculated for each standard solution after baseline correction and subtraction of the plastic material contribution (resulting from a variety of blank measurements). The mean peak height values (*I1003(sample)* − *I1003(blank), I311(sample)* − *I311(blank)*) along with the relative standard deviation values (RSD) were then calculated from the three replicate measurements performed (Table 8).

Taking into account the unusually high RSD values corresponding to the replicate measurements of TAZ standards, as well as the inability of detecting the characteristic TAZ peak for the last two standard solutions, the investigation of TAZ LoD value, at that stage of the research, was triggered. The visual evaluation method gave the LoD value between 1.80 and 2.00 mg/mL, which meant that TAZ LoQ value was in no case less than 5.50 mg/mL. Therefore, the therapeutic TAZ concentration range (from 3.33 to 5.00 mg/mL) lies under LoQ value, and TAZ API cannot be reliably quantified through the IV drip chamber. Thus, only on-line analysis of PIP API is possible.

- Linearity

**Figure 13:**
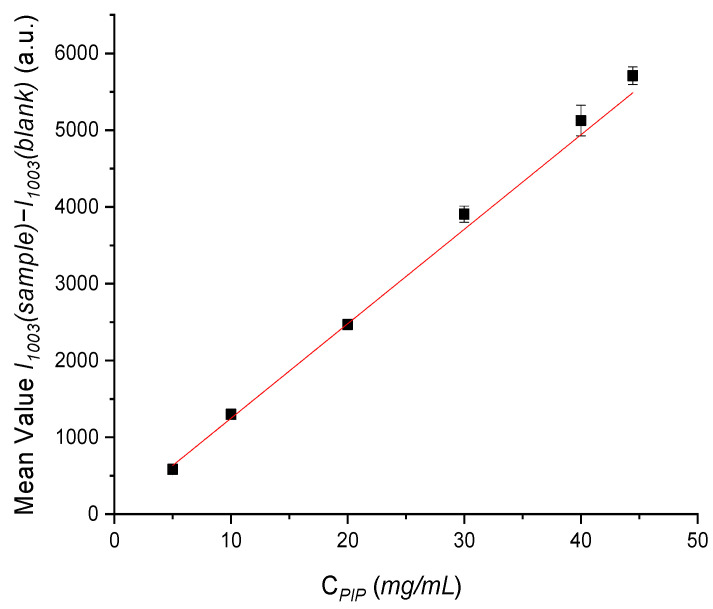
Calibration curve of PIP for standard solutions of liquid formulation placed in the drip chamber before administration, with concentration values ranging from 44.44 to 5.00 mg/mL.

The linearity of the method was confirmed by a six point concentration calibration curve for PIP, achieved by successive dilutions of an initial drug solution. The calibration curve was constructed using the intensity values (y axis) against concentration values (x axis) (Figure 13). Particularly, the value *I1003(sample)* − *I1003(blank)* was used against *C_PIP_*.

The respective equation describing the calibration line was:

*I1003(sample)−I1003(blank)* = (123.07±3.09) × *C_PIP_* + (17.76 ± 56.39), (R^2^ =0.997) (6)

- Intra- and Inter-day Precision

The intra-day (repeatability) and inter-day precision, tested over a period of 6 days, of the developed method were assessed using six determinations for each, at 100% of the test concentration, i.e., 40.00 mg PIP/mL.Using Equation (6), the repeatability study results gave: C = (41.32 ± 2.10) mg/mL and RSD = 5.08%. The inter-day precision gave: C = (40.16 ± 1.13) mg/mL and RSD = 2.81%.

- Accuracy

The accuracy of the method was assessed using nine determinations over three concentrations (three determinations for each concentration), covering the specified range. The accuracy test for PIP gave:

1. For 44.44 mg/mL, C = (46.24 ± 0.93) mg/mL, RSD = 2.00% and E_r_ = 4.05%.
2. For 20.00 mg/mL, C = (19.92 ± 0.07) mg/mL, RSD = 0.35% and E_r_ = 0.40% and
3. For 5.00 mg/mL, C = (4.60 ± 0.24) mg/mL, RSD = 5.14% and E_r_ = 8.00%.

- LoD and LoQ

Using Equation (6), and after many blank samples had been measured, PIP LoD was found to be 0.41 mg/mL, while LoQ was equal to 1.23 mg/mL. The LoD value was confirmed by visual evaluation method.

- Calibration Range

The concentration range used for the construction of the calibration line corresponds to values from 5.00 to 44.44 mg PIP/mL, within which analytical procedure provides an acceptable degree of linearity, precision, and accuracy. Additionally, this range includes the therapeutic concentration window, and ensures the total dissolution of the API present in the formulation intended for administration.

In order to test the performance of the calibration line once more, a formulation solution of 23.00 mg PIP/mL was prepared in order to be treated as unknown sample and it was measured in the same way. Using Equation (6), PIP concentration was found to be 22.19 ± 1.04 mg/ mL with a relative error of 3.51%.

Given the RSD values for PIP standards included in Table 8, three measurements for each sample would be needed in this case, thus the measurement process, once the drug has been led into the drip chamber, would not take more than 5 to 6 min.

## 3. Discussion

The aim of the present study was to develop an appropriate methodology for the simultaneous identification and quantification of piperacillin and tazobactam, two APIs that coexist in an intravenously administered formulation, with a mass ratio of 8:1. This methodology was developed in such a way to comply with the procedures of preparation and administration of IV drugs, so as to be applicable in hospitals. Therefore, it was intended to be non-invasive, aseptic, non-destructive for the sample, simple to be applied, quick and effective, and preferably of low cost. Thus, the proposed methodology could be immediately applied in order to eliminate errors relating to the administration of the wrong drug or the incorrect concentration. Currently applied techniques in hospitals for such purposes include chromatography with flow injection analysis (FIA), HPLC linked to UV/DAD (diode array detector), and spectroscopic apparatus equipped with UV/FT-IR. However, most of them are time-consuming, and some of the methods require considerable consumables, e.g., mobile phase, chromatographic columns, or sample preparation, which leads to exposure of staff to hazardous materials, especially in case of cytotoxic drugs [58]. Additionally, UV spectroscopy is not efficient when the co-examined materials have the same UV signatures and infrared spectroscopy is not appropriate for aqueous solutions’ analysis, as water considerably absorbs the infrared radiation [59]. So, the initial goal was approached through the utilization of Raman spectroscopy, while the formulation used was chosen due to the presence of two APIs with significant difference in their content in it, which itself rendered the desired analysis more complicated. Although the simultaneous identification and quantification of this pharmaceutical product has repeatedly been achieved, applying HPLC protocols, the whole procedure is not only time-consuming, destructive for the sample, and costly, but it also requires specialized staff. Additionally, it requires a large volume of solvents, thus excluding itself from the category of “green” techniques. UHPLC, on the other hand, takes only 5 min and few µL of sample to be applied, but the whole procedure still requires a mobile phase and a more specialized user. The above-mentioned drawbacks outweigh the high precision and the low limits of detection it offers.

The novel character of the approach presented here lies in that the developed methodology is fully compliant with the hospital routine procedures, two APIs in a single formulation are simultaneously analyzed, and both the solid and liquid formulation are measured. The proposed methodology involves testing procedures regarding not only the identity but also the composition of the formulation to be administered during three different preparation stages. More specifically, the drug analysis is possible either before or after the reconstitution process, as well as just before its administration to the patient, after it has been further diluted in the IV infusion bag.

The first part of the research was focused on the non-invasive (without removal of the substance out of the commercial glass bottle) qualitative and quantitative analysis of the initial dry powder of the formulation before reconstitution. For the identification of the solid drug at that stage, the spectrum of the solid formulation before reconstitution was compared with the spectra of pure APIs’ powder. All spectra were obtained by the use of the fiber optic probe of the Raman instrumentation, which was constantly in contact with the outer surface of the glass bottle, after the samples had been placed in the latter. Although the masking of the sample peaks due to glass intervention was extensive, and the similarity between the spectra of the formulation and the PIP API was great, due to the high content of the latter in the drug, the two APIs were identified and TAZ API was effectively detected using spectral differences. Consequently, a number of standard solid mixtures of the APIs were prepared and measured in the same way, with the mass ratio of PIP and TAZ in them ranging from 10% TAZ/90% PIP to 30% TAZ/70% PIP (targeting the mass ratio of 11% TAZ/89% PIP in the commercial formulation). A calibration curve was then constructed, through which the% *w*/*w* content of each API in the final product could be determined. The analytical procedure was also validated according to characteristics including linearity, intra- and inter-day precision, accuracy, LoD, LoQ, and calibration range.

The second part of the research was focused on the non-invasive qualitative and quantitative analysis of the liquid formulation inside the original glass bottle after reconstitution. For the identification of the liquid drug at that stage, the spectrum of the reconstituted formulation was compared to the spectra of pure APIs’ solutions. Once more, the measurements were performed by the aid of the fiber optic probe of the Raman instrumentation after the samples had been placed in the glass bottle. The extensive masking of sample peaks due to the nature of the methodology developed and the much higher content of PIP in the formulation did still make the detection of APIs, mainly in case of TAZ, more complex. Despite that, the identification was achieved once more. Standard formulation solutions were prepared, with nominal concentration values ranging from 266.67 to 5.00 mg/mL for PIP and from 60.00 to 7.00 mg/mL for TAZ. Calibration curves for each API separately were then constructed through polynomial regression method, while the relationship between the scattering signal and each API concentration was effectively expressed through quadratic equations. The whole analytical procedure was also validated according to characteristics including linearity, intra- and inter-day precision, accuracy, LoD, LoQ and calibration range. As it was further proved, on average, 15% of the initial dry powder of the formulation remained undissolved after the reconstitution process. The last-mentioned conclusion was the result of a study, which included the filtration of the reconstituted formulation, the quantitative analysis of the further diluted filtrate, as well as the characterization of the remaining powder on the filtering membrane.

The third part of the research was focused on the quantitative analysis of the liquid formulation, at-line and on-line, before administration to a patient. In this part, the sample was analyzed after it had been placed either in the drip chamber part of the IV administration set used to deliver the drug to the patient’s vein (on-line test) or in a special Raman optic cell (at-line test). The latter could be filled with 1–2 mL of the liquid drug after this minimum amount had been withdrawn from the infusion bag by the nursing staff and the aid of a syringe. In all cases, the measurements were performed using the fiber optic probe of the Raman instrumentation. After the ideal focus position of the laser beam had been selected, solutions of pure APIs and the formulation, along with standard solutions of the latter, were placed in the sample holders in order to be measured. In case the Raman optic cell was used, the concentration values of the standards ranged from 44.44 to 5.00 mg PIP/mL and from 5.56 to 3.75 mg TAZ/mL respectively, values that belong to the therapeutic concentration range. One calibration curve for each API was constructed. In case the IV drip chamber was used, the quantification of PIP API only could be reliable, due to the fact that the therapeutic TAZ concentration range (from 3.33 to 5.00 mg/mL) lied under its LoQ value. The analytical procedure developed for the on-line determination of PIP API was also validated according to characteristics including linearity, intra- and inter-day precision, accuracy, LoD, LoQ and calibration range.

Finally, the spectral image, as well as the LoD values achieved for both APIs, obtained by each one of the three methodologies suggested (commercial glass bottle, drip chamber of the IV set, and the Raman optic cell, see in Table 9) are representative of the quality and efficacy of each one applied. Particularly, either the glass material of the commercial bottle or the polyethylene of which the drip chamber is made leads to extensive masking of analytes’ peaks, and the LoD values achieved by these two methods are almost identical. On the other hand, the quartz material of which the Raman cell is made allows the detection of sample peaks in a wider spectral window; thus, the LoD value determined in this case is almost one-third that of the other sample holders. This is due to the fact that the quartz material of the outer cell surface is thinner compared to the glass and polyethylene surface of the remaining two sample holders used, and the mirror presence in the back part of it triggers the reflection of the radiation beam; thus, the enhancement of the signal intensity is achieved, and the sensitivity of the methodology was enhanced. The characteristics of the analytical alternatives presented in this study are summarized in the following comparative table.

## 4. Materials and Methods

In the present study, pure piperacillin and tazobactam APIs, were purchased in the form of sodium salts from Glentham Life Sciences (Corsham, Wiltshire, UK), which is a UK-based supplying company. The generic formulation used was Zobactam^®^ (Vocate Pharmaceuticals S.A., Athens, Greece), a combination of piperacillin and tazobactam in a mass ratio of 8:1, respectively. The formulation was in the form of powder for injectable solution with no excipients and had been supplied by Aenorasis S.A., a Greece-based medical equipment company (Athens, Greece). In order for complete reconstitution of 4.5 g of the drug to be achieved, 20 mL of sodium chloride solution 0.9% *w*/*v* (Vioser S.A. Parenteral Solutions Industry, Trikala, Greece) were used. Further dilution of the reconstituted formulation resulted in a solution of a total volume between 100 and 150 mL. The clinically relevant concentration values of PIP and TAZ in the final liquid formulation ready for administration ranged from 26.67 to 40.00 mg PIP/mL and from 3.33 to 5.00 mg TAZ/mL, respectively, depending on the separate needs of each patient.

### 4.1. Preparation of Standard Mixtures

Pure APIs were used to prepare five dry mixtures. The mass ratio of TAZ:PIP ranged from 30:70 to 10:90. Using an analytical balance (Kern Inc., Grove City, OH, USA, ABJ 220-4NM) and depending on the desired ratio, an appropriate amount of each API was obtained in order a total mass of 100 mg of the standard mixture to be prepared (e.g., for the dry mixture 20:80, 20 mg TAZ and 80 mg PIP were obtained). Each mixture was placed in a special plastic container (NALGENE^®^, Rochester, NY, USA) with a magnetic rod and was homogenized by placing the latter on a magnetic stirrer (HANNA instruments, HI 190M (Woonsocket, RI, USA)) for 5 min.

### 4.2. Preparation of Standard Solutions

The reconstitution of the initial solid formulation took place twice by adding saline solution 0.9% through the rubbery cap of the commercial glass bottle using a syringe. Except from constant shaking of the bottle for 10 min (according to the instruction leaflet of the drug), Vortex (IKA^®^, Staufen, Germany, MS2, Minishaker) and an ultrasonic bath (Branson Ultrasonics, Danbury, CT, US, 2510E-MT) at a frequency of 60 HZ were also used for about 30 s and 15 min, respectively. According to calculations, the solution after the first reconstitution process had a nominal concentration of 266.67 mg PIP/mL, and that after the second reconstitution process had a nominal concentration value of 60.00 mg TAZ/mL. By the method of successive dilutions inside the commercial glass bottle, another 10 PIP standard solutions were prepared, and their concentration values ranged from 200.00 to 5.00 mg/mL, whereas the remaining 9 TAZ standard solutions prepared had concentration values ranging from 60.00 to 7.00 mg/mL. The solvent was added using automatic pipettes (Labnet International Inc., Edison, NJ, USA, Biopette^TM^, Autoclavable Pipettes).

### 4.3. Experimental Methodology for the Dissolution Test after Reconstitution

The standard solution under test was filtered using a filtering device and a 0.22 μL nitrocellulose membrane (Merck Millipore Ltd., Burlington, MA, USA). Then, the filtrate was diluted five times, while the diluent was the solution of NaCl 0.9%. The filtering membrane was finally kept in a glass container (Bormioli Rocco, Fidenza, Italy) with dehydrating material for 24 h in order the remaining moisture to be removed.

### 4.4. Methodology of Raman Spectra Acquisition

Raman spectra were obtained using a portable Raman spectrometer combined with an optical microscope (i-Raman Plus^®^, B&W Tek Inc., Newark, DE, USA). The instruments’ nominal power was equal to 350 mW, and the laser beam used was at 785 nm.

#### 4.4.1. Method of High Reflectiveness Gold-Coated Slide

Pure APIs in their solid or liquid form, as well as the formulation, were measured by placing a small amount of the sample onto a high reflectiveness gold-coated slide (EMF Corporation, Ithaca, NY, USA). That slide was coated by a 50 Å titanium layer and, above this, a 1000 Å gold layer. The latter gives the slide high reflectiveness. The sample was placed thereon, and in case it was in its solid state, a low pressure was applied on top of it by the aid of a spatula, in order to make its surface flat and for a better signal-to-noise ratio to be achieved in the spectrum obtained. Each measurement was a result of 5 accumulations in the spectral region from 0 to 2814 cm^−1^, while the accumulation time was 20 s and the laser power equal to 210 mW.

#### 4.4.2. Method of Glass Bottle

Pure APIs in their solid or liquid form, their commercial mixture before reconstitution, as well as standard mixtures and standard solutions under test were analyzed without removing them from the glass bottle. Their spectra were obtained using the fiber optic probe of the instrument. Particularly, by properly adjusting the distance between the fiber optic probe and the outer surface of the glass bottle (the probe touched the outer surface of the glass), the laser beam was focused inside the bottle, thus enabling the capture of the spectral image of the sample. Each sample was measured 6 times from 6 differerent areas of the bottle, which had been marked and defined on it. In this way, the glass-induced variability, which could have had an effect on the results, would be eliminated. The commercial glass bottle had a diameter of 4.60 cm, while the mean thickness value coming from the six measuring positions was equal to 2.40 mm with a standard deviation value of 0.19 mm. Each measurement was a result of 5 accumulations in the spectral region from 0 to 2814 cm^−1^, while the accumulation time was 20 s and the laser power equal to 280 mW.

#### 4.4.3. Method of Drip Chamber

An intravenous gravity administration set (Primary PLUM^TM^ Set 14000, Hospira Inc., Lake Forest, IL, USA), with a length value of 272 cm and liquid capacity of 19 mL, which is used in a hospital workflow so the drug reaches a patient’s vein, was filled with each of the standard solutions under test. Part of the IV set was the drip chamber, which, in this case, was the sample holder for the Raman spectra acquisition in the methodology developed. The main parts of the IV set, as well the drip chamber, were made of polyethylene. Each measurement was a result of 5 accumulations in the spectral region from 0 to 2814 cm^−1^, while the accumulation time was 20 s and the laser power equal to 280 mW. As in the method of the glass bottle, the spectra were obtained using the fiber optic probe of the instrument, while the distance between the probe and the outer surface of the drip chamber played an important role in the quality of the received spectra.

#### 4.4.4. Method of Raman Optic Cell

In this case, solutions of the liquid formulation under test were transferred in a Raman optic cell, which had a mirror placed on the back part of it (Hellma GmbH & Co. KG, Müllheim, Germany). The cell was made of synthetic quartz (Quartz Suprasil^®^ 300), its thickness, which is the same as the optical path length, was equal to 5 mm, and it required 1.7 μL to be filled. Both the front quartz and the back mirror wall of the cell had a thickness value of 1 mm. Each measurement was a result of 5 accumulations in the spectral region from 0 to 2814 cm^−1^, while the accumulation time was 20 s and the laser power equal to 280 mW. As in the method of the glass bottle, the spectra were obtained using the fiber optic probe of the instrument, while the distance between the probe and the outer surface of the cell played an important role in the quality of the received spectra.

### 4.5. Spectra Processing

Spectral data were processed using Origin (OriginPro8, OriginLabPro^®^, Northampton, MA, USA). The OriginPro8 integration method of measuring the absolute height (or intensity) of selected peaks was used. In the case of solid samples, different combinations of fingerprint APIs’ peaks were tested so as to assess the potential to quantify the level of APIs present. After trials, the selected peaks were at 517 cm^−1^, which was the result of both APIs’ presence and the characteristic PIP peak at 1003 cm^−1^. The baseline corresponding to the first peak ranged from 500 to 550 cm^−1^, while the baseline for the second peak ranged from 987 to 1019 cm^−1^. As for the liquid samples, either they were placed in the glass bottle or in the drip chamber and the mirror cuvette, and the peaks selected for the quantification were characteristic of each API. As before, the PIP peak used was at 1003 cm^−1^, and the respective baseline ranged from 987 to 1019 cm^−1^, while for TAZ, the peak used was found at 311 cm^−1^, and its baseline ranged from 295 to 325 cm^−1^.

## 5. Conclusions

The proposed methodology as a whole has the potential to be applied to intravenously administered formulations, for either on-line or at-line analysis, given that it can comply with the preparation and administration procedures followed. The important advantages Raman spectroscopy gathers, such as the fast analytical response, the absence of sample preparation necessity, the non-invasive analysis nature, the negligible maintenance costs, the reassurance of operator safety, the limited waste produced, the suppression of consumables, and the reduction in staff training cost, render it a valuable analytical tool. In terms of a robust transportable system design equipped with deported probes to handheld devices, large libraries of spectral data, and an inter-system calibration (quantitative algorithms), the gap between experimental setup and clinical implementation could be bridged.

## Figures and Tables

**Table 1:** Characteristic peaks of pure APIs and their attribution to vibration modes according to bibliography.

API	Wavenumber (cm^−1^)	Vibration Mode
PIP	406.0	Carbonyl group (CO) bending
	890.0	C-H bending
	1003.0, 1032.0	Aromatic C-C-C bending
	1397.6	N-H stretching
	1456.0	C-C stretching
	1486.3	N=H bending
	1587.1, 1603.8, 1683.9	C=C stretching
TAZ	625.4	C-S stretching (Sulfone Ring)
	1231.1	N=N stretching (Triazole Ring)
	1290.4	Triazole Ring Bending
	1400.6	CO_2_^−^ stretching
	1487.7	C=C stretching (Triazole Ring)
	1780.9	Carbonyl group (CO) stretching

**Table 2:** Spectral differences between PIP and formulation Raman spectra obtained from either solid or liquid samples of the substances on a g.slide. TAZ presence accounts for such differences.

Wavenumber (cm^−1^)	Observation
311.1	Intensity enhancement (solid state)/ detection of TAZ peak (liquid state, in this case PIP peak is not detected at the certain position)
517.0	Intensity enhancement and change in the morphology of the broad PIP peak in area A
625.4	Detection of TAZ shoulder
875.1	Intensity enhancement and change in intensity ratio between neighboring PIP peaks in area B
1321.9	Intensity enhancement and shift of the relatively broad, weak PIP peak in area C

**Table 3:** Mean peak height ratio, *I*(517)/*I*(1003), and relative standard deviation (RSD) values corresponding to the six measurements performed, for each one of the standard mixtures placed in the commercial glass bottle.

% Mass Ratio TAZ/PIP	*I*(517)/*I*(1003)	RSD%
30:70	0.145	6.207
20:80	0.121	8.264
15:85	0.102	4.902
12.5:87.5	0.099	7.071
10:90	0.084	5.952

**Table 4:** Mean peak height values *I1003(sample)* − *I1003(blank)* and *I311(sample)* − *I311(blank)* and respective relative standard deviation (RSD) values corresponding to the six measurements taken from the six different marked areas on the commercial glass bottle.

API	C (mg/mL)	*I*(sample) − *I*(blank) (a.u.)	RSD%
PIP (1003.0 cm^−1^)	266.67	29028.38	8.24
	200.00	24576.15	9.16
	177.77	21479.97	6.90
	133.33	16696.92	6.62
	88.89	11867.34	8.07
	44.44	5848.70	8.78
	40.00	5654.34	8.77
	30.00	3981.94	4.11
	20.00	2634.56	3.81
	10.00	1350.39	4.60
	5.00	655.83	7.15
TAZ (311.1 cm^−1^)	60.00	3001.38	6.93
	50.00	2694.73	14.45
	40.00	2332.64	11.06
	33.33	2039.69	7.66
	25.00	1662.18	8.10
	22.22	1420.92	5.49
	16.67	1105.93	6.21
	11.11	840.31	10.09
	9.00	630.67	6.82
	7.00	494.35	9.46

**Table 5:** Remarks on the position of the focused beam regarding the Raman optic cell, in correlation with the respective spectrum received. The peak mentioned in the remarks is the characteristic PIP peak at 1003 cm^−1^.

Focus Position	REMARKS
A or F	front quartz surface or back mirror surface, the peak is detectable but quite weak especially in case of position A
B or E	at a depth of 1 or 4 mm (very close to the front quartz or the back mirror surface), the peak signal is stronger than before but is not as strong as it could be
C	at a depth of 2 to 2.5 mm (approximately in the middle of the radiation path), the peak signal is close to its maximum value
D	at a depth of 4 mm (closer to the back mirror than to the front quartz surface), the peak signal is the maximum that could be obtained

**Table 6:** Mean peak height values *I1003(sample)* − *I1003(blank)* and *I311(sample)* − *I311(blank)* and respective relative standard deviation (RSD) values corresponding to the three measurements obtained from the samples after they had been placed in the Raman optic cell.

API	C (mg/mL)	*I*(sample) − *I*(blank) (a.u.)	RSD%
PIP (1003.0 cm^−1^)	44.44	13800.36	1.77
	40.00	12062.34	1.60
	30.00	9463.25	0.80
	20.00	6354.26	0.75
	10.00	3084.76	1.51
	5.00	1561.74	1.66
TAZ (311.1 cm^−1^)	5.56	899.10	5.47
	5.00	761.14	9.22
	4.80	746.78	4.52
	4.20	626.54	4.08
	3.75	593.24	6.42

**Table 7:** Remarks on the focus position of the laser beam on the drip chamber of the IV set, in correlation with the respective spectrum received. The peak mentioned in the remarks is the characteristic PIP peak at 1003 cm^−1^.

Focus Position	REMARKS
A	outer front plastic surface, the peak is very weak
B	at a depth of 0.45 cm (close to the front plastic surface), the signal is stronger than before but not the maximum
C	at a depth of 0.9 to 1 cm (approximately in the middle of the radiation path), the peak signal is the maximum that could be obtained
D	at a depth of 1.35 cm (closer to the back than to the front plastic surface), the peak signal is close to its maximum value
E	at a depth larger than 1.60 cm (close to the back plastic surface), the signal is not as strong as it could be

**Table 8:** Mean peak height values *I1003(sample)* − *I1003(blank)* and *I311(sample)* − *I311(blank)* and respective relative standard deviation (RSD) values corresponding to the three measurements obtained from the samples after they had been placed in the drip chamber of the IV set.

C (mg/mL)		*I*(sample) − *I*(blank) (a.u.)		RSD%	
PIP	TAZ	1003.0 cm^−1^	311.1 cm^−1^	1003.0 cm^−1^	311.1 cm^−1^
44.44	5.56	5708.64	349.73	2.00	5.77
40.00	5.00	5126.35	356.17	3.90	13.84
30.00	3.75	3905.66	185.09	2.69	26.59
20.00	2.50	2468.87	161.20	0.35	20.25
10.00	1.25	1298.05	not detected	1.38	not detected
5.00	0.62	583.98	not detected	4.99	not detected

**Table 9:** Stage and nature of analysis, the measurement recording time, the respective calibration curve/equation, LOD, and precision control results, regarding the analytical alternatives–methodologies suggested here, i.e, measurement through the commercial glass bottle, the drip chamber part of the IV set, or a Raman optic cell.

Method/ Container	Analysis Stage/ Nature/ Recording Time	Calibration Curve/Equation		LoD	Precision Control (C_unknown_, E_r_)	
**Glass Bottle**	Before Reconstitution/ Non-invasive/ ≅10 min	Linear Curve/ $\frac{I\;\left(517\;{\mathrm{cm}}^{-1}\right)}{I\;\left(1003\;{\mathrm{cm}}^{-1}\right)}$= (0.192 ± 0.025)$\times \left(\frac{100}{C_{PIP}}\right)$+ (−0.125 ± 0.029), (R^2^ = 0.953)		TAZ	PIP	TAZ
				3.01% *w/w*	(90.17 ± 1.95)% *w/w*, 1.31%	(90.17 ± 1.95)% *w/w*, 1.31%
	After Reconstitution/ Non-invasive/ ≅10 min	PIP	Polynomial Curve / *I1003(sample)* − *I1003(blank)* = (−0.10 ± 0.02)*C_PIP_ ^2^+ (138.94 ± 2.88)*C_PIP_ + (−40.82 ± 31.46), (R^2^ = 0.999)	0.60 mg/mL	(143.52 ± 10.52) mg/mL, 5.58%	
					(27.05 ± 2.02) mg/mL, 6.08%	
		TAZ	Polynomial Curve / *I311(sample)* − *I311(blank)* = ( −0.40 ± 0.06)*C_TAZ_^2^+ (73.99 ± 3.07)*C_TAZ_ + (1.35 ± 28.86), (R^2^ = 0.997)	1.85 mg/mL	(20.42 ± 1.42) mg/mL, 7.47%	
					(3.30 ± 0.75) mg/mL, 8.32%	
**Drip Chamber**	Before Administration/ Non-invasive/ ≅5 min	PIP	*I1003(sample)* − *I1003(blank)* = (123.07±3.09)*C_PIP_ + (17.76 ± 56.39), (R^2^ =0.997)	0.41 mg/mL	(22.19 ± 1.04) mg/mL, 3.51%	
**Raman Cell**	Before Administration/ Invasive or not/ ≅5 min (3 repeats) Or ≅1.5 min (1 repeat)	PIP	*I1003(sample)* − *I1003(blank)* = (314.30 ± 3.81)*C_PIP_ + (−8.38 ± 52.20), (R^2^ = 0.999)	0.24 mg/mL	(22.46±0.60) mg/mL, 2.45%	
		TAZ	*I311(sample)* − *I311(blank)* = (173.05 ± 19.13) *C_TAZ_ + (−84.21 ± 85.82), (R^2^ = 0.965)	0.94 mg/mL	(3.05±0.11) mg/mL, 6.27%	

## Data Availability

Data is contained within the article.

## Sample Availability

Samples of pure APIs, PIP and TAZ, are available from the authors.

## Appendix A

Appendix A is a section containing the mathematical proof of how the final equation of the calibration curve (Figure 4, Equation (1)) for the quantification of PIP and TAZ in the solid formulation before reconstitution was developed. Spectral observations arising from powder spectra of both pure active substances and the commercial formulation (Figure 2 and Figure 3) confirmed that peak at 517 cm^−1^ is a result of not only PIP but also TAZ presence. Thus, signal intensity at that position is expected to be given from the equation:

(A1) $$I\;\left(517\;{\mathrm{cm}}^{-1}\right)=k_{1}\times C_{PIP}+k_{2}\times C_{TAZ},$$

where $k_{1}$ and $k_{2}$ are standards correlating the value $I\;\left(517\;{\mathrm{cm}}^{-1}\right)$ with $C_{PIP}$ and $I\;\left(517\;{\mathrm{cm}}^{-1}\right)$ with $C_{TAZ}$, respectively.

Due to the fact that the peak at 1003 cm^−1^ is characteristic of piperacillin, its intensity is expected to be given from the equation:

(A2) $$I\;\left(1003\;{\mathrm{cm}}^{-1}\right)=k_{3}\times C_{PIP},$$

where $k_{3}$ is a standard correlating the value $I\;\left(1003\;{\mathrm{cm}}^{-1}\right)$ with $C_{PIP}$.

If (A1) is divided by (A2), then it turns out that:

(A3) $$\frac{I\;\left(517\;{\mathrm{cm}}^{-1}\right)}{I\;\left(1003\;{\mathrm{cm}}^{-1}\right)}=\frac{k_{1}}{k_{3}}+\;\frac{k_{2}}{k_{3}}\times \frac{C_{TAZ}}{C_{PIP}}$$

Given that the formulation consists exclusively of the APIs, it is valid that:

(A4) $$C_{TAZ}=100-C_{PIP},$$

Finally, combining (A3) with (A4), a new equation is formed, where:

(A5) $$\frac{I\;\left(517\;{\mathrm{cm}}^{-1}\right)}{I\;\left(1003\;{\mathrm{cm}}^{-1}\right)}=\frac{k_{1}-k_{2}}{k_{3}}+\;\frac{k_{2}}{k_{3}}\times \frac{100}{C_{PIP}}$$
